# A 3-month-delayed treatment with anatabine improves chronic outcomes in two different models of repetitive mild traumatic brain injury in hTau mice

**DOI:** 10.1038/s41598-021-87161-7

**Published:** 2021-04-12

**Authors:** Alexander Morin, Benoit Mouzon, Scott Ferguson, Daniel Paris, Nicole Saltiel, Mackenzie Browning, Mike Mullan, Fiona Crawford

**Affiliations:** 1 The Roskamp Institute, 2040 Whitfield Ave., Sarasota, FL 34243 USA; 2 The Open University, Milton-Keynes, UK; 3 James A Haley Veterans Administration, Tampa, FL USA

**Keywords:** Regeneration and repair in the nervous system, Neurodegeneration

## Abstract

To date, an overwhelming number of preclinical studies have addressed acute treatment in mild TBI (mTBI) and repetitive mTBI (r-mTBI), whereas, in humans, there often exists a significant time gap between the injury and the first medical intervention. Our study focused on a delayed treatment with anatabine, an anti-inflammatory compound, in hTau mice using two different models of r-mTBI. The rationale for using two models of the same impact but different frequencies (5 hit mTBI over 9 days and 24 hit mTBI over 90 days) was chosen to address the heterogeneity of r-mTBI in clinical population. Following the last injury in each model, three months elapsed before the initiation of treatment. Anatabine was administered in drinking water for 3 months thereafter. Our data demonstrated that a 3-month delayed treatment with anatabine mitigated astrogliosis in both TBI paradigms but improved cognitive functions only in more-frequently-injured mice (24 hit mTBI). We also found that anatabine decreased the phosphorylation of tau protein and NFκB, which were increased after r-mTBI in both models. The ability of anatabine to suppress these mechanisms suggests that delayed treatment can be effective for clinical population of r-mTBI. The discrepancy between the two models with regard to changes in cognitive performance suggests that r-mTBI heterogeneity may influence treatment efficiency and should be considered in therapeutic development.

## Introduction

According to epidemiological studies, 80% of all TBIs are mild^1,2^. They result from acceleration-deceleration forces that are commonly triggered during motor vehicle accidents, falls, collisions in contact sports and injuries in military operations^1^. The latter two represent the groups—athletes and the military—which are most susceptible to repeated exposure to mTBI. Compared to single injuries, such repetitive mTBI (r-mTBI) may lead to long-term functional and neuropathological deficits and increased risk of neurodegenerative diseases^3,4^.

To date, there is no FDA-approved treatment available for r-mTBI. A major obstacle to developing an efficient treatment is the high heterogeneity of r-mTBI etiology, and of the pathological response, including axonal injury, inflammatory response, oxidative stress, neurovascular damage, accumulation of tau, apoptosis, and other processes. Of all aspects of r-mTBI pathobiology, neuroinflammation is perhaps the most consistent, reported in human and preclinical studies and linked to chronic TBI and Chronic Traumatic Encephalopathy (CTE)^4–7^. Existing drugs with anti-inflammatory action have typically been tested in moderate and severe models of TBI^8^, with treatment options in r-mTBI being relatively understudied. However, even those drugs that have shown efficacy in preclinical models of moderate and severe TBI (e.g. progesterone, statins, minocycline) failed to resolve TBI-associated neurological outcomes in clinical trials^9–11^. More detailed preclinical studies involving investigations of relevant dosing, timing of administration etc. would better support translation. In r-mTBI animal models, compared to human cases, inaccurate replication of the time gap between the last injury and the first medical intervention may undermine successful translatability. While the majority of the clinical population do not seek medical attention until months or years after the concussion, only 10% of preclinical TBI papers assessed functional outcomes > 2 months post injury, even less so for mTBI and r-mTBI^1,12^. Despite growing attempts, studies investigating potential treatment at delayed time points, which are representative of the human population, are scarce. Hence, the first aim of the studies in this paper was to address the effects of an anti-inflammatory treatment in r-mTBI mice administered at a delayed timepoint—3 months post last injury.

**Figure 1:**
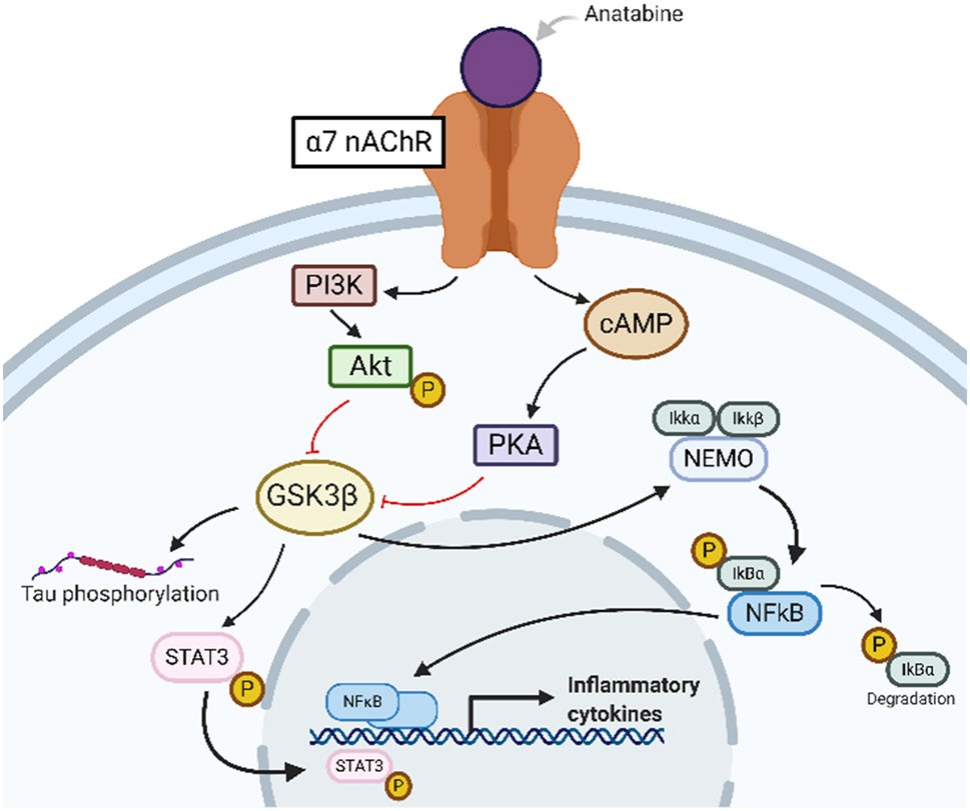
Anatabine acts as an agonist of α7 nAChR which activates PI3K-Akt and cAMP-PKA signaling pathways. Both Akt and PKA phosphorylate GSK3β at Ser9 which results in its inhibition. Inactivation of GSK3β leads to a decrease of phosphorylation of tau and of STAT3, preventing the latter from translocating into the nucleus and initiating transcription of inflammatory cytokines. Similarly, phosphorylated GSK3β fails to phosphorylate the Ikk/NEMO complex which is responsible for activation of NFκB.

We have previously demonstrated the anti-inflammatory effects of the naturally occurring compound anatabine in our models of Alzheimer’s disease (AD)^13,14^ and experimental autoimmune encephalomyelitis (EAE)^15^, and which showed promise for TBI treatment in an earlier study^16^. Anatabine acts as an agonist of nicotinic receptors (nAChR) and, in particular, is an agonist of the α7 subtype^17^ and inhibits both the signal transducer and activation of transcription-3 (STAT3) and nuclear factor kappa-light-chain enhancer of activated B cells (NFκB) activation^14^ (Fig. 1). Our team demonstrated that therapeutic properties of anatabine extend beyond its anti-inflammatory activity and also include a decrease in tau phosphorylation at multiple epitopes (pThr231, pSer396/404, pSer202) in Tg APPsw and P301S mouse models of AD^13,14^. We also tested the effects of anatabine in our well-characterized model of repetitive mTBI (r-mTBI) where mice received 5 mild closed-head injuries (5r-mTBI) over 9 days (48 h interval between the hits)^18–20^. During a 9-month treatment with anatabine, starting immediately after the last injury, anatabine improved cognitive deficits at 6 months and decreased inflammation at 9 months post-TBI^16^. At 9 months after r-mTBI/sham in this study we performed a cross-over, so that the previously assigned Vehicle mice began treatment with anatabine at 9 months post-last injury. We found that such delayed treatment also decreased microgliosis in the corpus callosum, evident at 18 months post-TBI. These data demonstrate, at least preclinically, that late administration of anatabine can be effective at mitigating against the negative sequelae of mTBI, which is encouraging for mTBI patients who typically do not seek medical attention at acute or subacute timepoints after injury.

In clinical populations, accumulation of phosphorylated tau is now a recognized hallmark of chronic pathology of r-mTBI. In fact, most observations of tauopathy resulting from brain injuries are reported in contact sport players who sustain multiple concussions throughout their career^4,21^. Thus, to replicate the possibly beneficial effects of anatabine on tau, we decided to utilize hTau mice, which express all 6 isoforms of human tau^22^, and in which we have previously demonstrated r-mTBI-dependent increases in tau phosphorylation and oligomerization^20,23,24^.

The second aim of our study was to further model and explore the heterogeneity of TBI in the human patient population. We have developed several different injury paradigms, using the same midline mTBI first described by Mouzon et al.^18^. The two paradigms used in this study were a 5 hit model (5r-mTBI), 5 hits over 9 days with a 48 h interval between hits^18^, and a 24 hit repetitive mTBI model (24r-mTBI) in which mice receive 2 hits per week for 3 months^23^.

To address the anticipated delay in treatment administration to human r-mTBI patients, and increase the translational relevance of our study, we administered a 3 month treatment with the anti-inflammatory anatabine beginning at a delayed timepoint, 3 months after the last injury, in these two models of r-mTBI (5r-mTBI and 24r-mTBI).

## Materials and methods

### Animals

Male and female hTau mice 12–14 weeks old (weight 19–25 g) were sourced from Jackson Laboratories (Bar Harbor, ME). The animals were housed under standard laboratory conditions (14-h light/10-h dark cycle, 23 ± 1 °C, 50 ± 5% humidity) with free access to food and water. All procedures were carried out under Institutional Animal Care and Use Committee (IACUC) approval and in accordance with the National Institutes of Health Guide for the Care and Use of Laboratory Animals. The study was approved by the institutional review board of the Roskamp Institute.

### Experimental groups and study design

**Figure 2:**
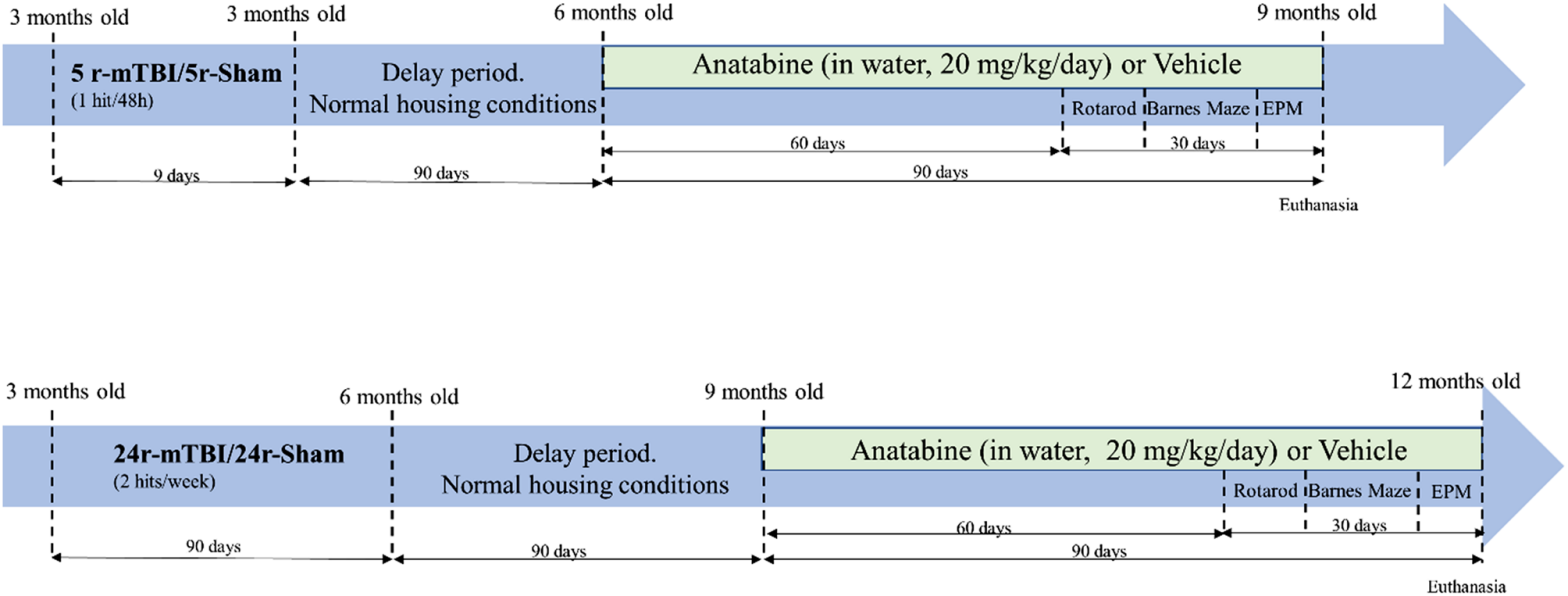
Study design.

The study was comprised of 2 cohorts of 3 months old hTau mice that received either 5 r-mTBI/sham (n = 34) or chronic-r-mTBI/sham (24r-mTBI, n = 38). In the 5r-mTBI cohort, TBI mice received 5 injuries over 9 days with a 48-h inter-concussion interval^18–20,25–28^. In the 24r-mTBI cohort, mice received a total of 24 injuries—2 injuries each week (with a 3–4 day inter-concussion interval) for the duration of 3 months (Fig. 2)^23^. The timelines chosen for these two groups were designed in order to match TBI mice by age at first injury (3 months old). This resulted in the mismatch of animals’ age at euthanasia (5r-mTBI—9 months; 24r-mTBI—12 months). Each cohort included four groups: sham-vehicle, r-mTBI-vehicle, sham-anatabine, r-mTBI-anatabine. The distribution of mice between the groups in each cohort is shown in Table 1. Each group included both male and female mice.

**Table 1:** Distribution of mice in the experimental groups (M-males, F-females).

	5r-mTBI	24r-mTBI
Sham-vehicle	8 (4M, 4F)	9 (5M, 4F)
r-mTBI-vehicle	10 (5M, 5F)	11 (5M, 6F)
Sham-anatabine	7 (4M, 3F)	8 (5M, 3F)
r-mTBI-anatabine	9 (6M, 3F)	10 (6M, 4F)

For sham/mTBI procedures, all animals underwent anesthesia with 1.5 mL/min of oxygen and 3% isoflurane for 3 min on the heating platform to prevent hypothermia. TBI mice continued to be kept on a heating pad during the injury procedure to prevent hypothermia. The head of each animal was fixed in a stereotaxic frame, and a 5 mm blunt metal impactor was positioned midway to the sagittal suture. The injury was triggered at 5 m/s velocity and 1.0 mm depth, with a dwell time of 200 ms, using a myNeuroLab controller device (Impact One Stereotaxic Impactor, Richmond, IL)^18^. All TBI mice experienced short-term apnea (< 20 s) and showed no skull fractures. All animals (sham and TBI) recovered from anesthesia on a heating pad and then returned to their cages with water and soft food access.

Regardless of the paradigm to which mice were assigned, after the last injury/sham procedure, mice were kept under normal housing conditions for 3 months. One week prior to the initiation of treatment, acclimation and baseline levels for Rotarod were recorded. No other manipulations on mice were conducted. After a 3-month period, anatabine (racemic anatabine, Anthem Biosciences, Bangalore, India) was administered in drinking water to mice assigned to the Sham-Anatabine and TBI-Anatabine groups at a concentration calculated to deliver 20 mg/kg/day. The volume was calculated based on the average weight of mice prior to treatment (25.5 g) and a previously calculated water consumption volume for C57BL/6 mice of 7.4 mL/30g^29^. The anatabine solution was changed weekly for 3 months. Placebo mice received regular water. Behavior tests started on day 61 after the initiation of treatment and included Rotarod, Barnes Maze, and Elevated Plus Maze as shown in the Study Design in Fig. 2. Euthanasia was performed 90 days after the initiation of treatment. Behavioral and histological assessments were carried out by researchers blinded to the experimental group assignments.

### Behavior

Behavior was assessed during the last month of treatment and included Rotarod, Barnes Maze (BM) and Elevated Plus Maze (EPM) (Fig. 2). Briefly, Rotarod acclimation and baseline levels were recorded during a delay period before the initiation of treatment. Acclimation included 3 trials for 5 min without acceleration while the baseline trial was performed using acceleration mode 5 to 50 rpm. Rotarod assessment started on day 61 after the beginning of anatabine treatment and was performed every other day during a 7-day trial. All data is presented as normalized to group baselines. The percentage change in latency between the groups was calculated using the average values for latency on day 7 by utilizing the formula: (100% × (latency2 − latency1)/latency1).

Each animal underwent 3 trials for 5 min with 5 min interval rest. After the final day of rotarod testing, cognitive function was evaluated using the BM. For a period of 6 days, four trials were given per day, with mice starting from one of four cardinal points on each trial. On the 7th day, a single probe trial lasting 60 s was performed with the mouse starting from the center of the maze and the target box removed. The percentage change in latency/distance between the groups was calculated using the average values for latency or distance on day 7 by utilizing the formula: (100% × (value2 − value1)/value1).

Disinhibition behavior was assessed using the EPM on the day prior to euthanasia. Here, disinhibition refers to risk-taking behavior, by which mice tend to spend more time in open vs closed space. Mice were placed in the middle of the plus-shaped maze elevated 80 cm above the ground with 2 open and 2 closed arms perpendicular to each other in a brightly lit room. Animal movement was recorded during a 5 min trial. Each animal underwent only one trial. The center point of the mouse, determined by Ethovision XT14 tracking, was used to determine which arm of the maze the mouse occupied at any given time. The ratio of time spent in open vs closed arms to total trial duration was calculated and these ratios were compared between the cohorts.

### Tissue processing and immunofluorescence

At 6 months post last mTBI/sham injury, mice were anesthetized with isoflurane and perfused transcardially with phosphate-buffered saline (PBS) with heparinized PBS, Ph-7.4. Brains were removed and post fixed in a solution of 4% paraformaldehyde at 4 °C for 48 h, dehydrated in graded ethanol solutions, cleared in xylene, and embedded in paraffin. Serial sections (6 µm thick) were cut onto positively charged glass slides and boiled in citrate buffer (10 mM pH 6.0) for antigen retrieval, followed by incubation with the following antibodies: GFAP/NFκB/RZ3*.* Fluorescent staining was performed with the antibodies for astroglial marker GFAP (7857983, Aves Labs), NFκB (3034, Cell Signaling) and phosphorylated tau at Thr231 (RZ3, Peter Davies). Following rehydration, antigen retrieval was performed by heating slides in citric acid buffer for 7 min in a microwave oven. Next, slides were washed with PBS and transferred to a Sudan Black solution for 15 min to prevent autofluorescence. Slides were then blocked for 1 h with 10% donkey serum solution in PBS, and primary antibodies for GFAP (1:500), NFκB (1:500) and RZ3 (1:500) were applied overnight. On the next day, secondary antibodies AlexaFluor488 (A21202, Life Technologies) and AlexaFluor647 (ab175477, Abcam) were applied. Slides were mounted with ProLong Gold Antifade 4′,6-diamidino-2-phenylindole (DAPI) Mount. Imaging was performed using a confocal microscope (LSM 800 Zeiss) at × 20 magnification. Quantification of the fluorescent images was performed using LSM 800 Zeiss and the intensity of fluorescence was measured for each antibody. The same area of interest was applied to each image and the background signal was subtracted from the obtained values. Quantitative analysis of colocalization of NFkB and DAPI was performed by calculating Pearson’s coefficient (PC) of selected area. PC range is between − 1 and 1, where − 1 indicates negative correlation, 0—lack of colocalization, and 1—positive correlation (colocalization).

### Statistical analysis

All experimental data were analyzed using JMP Pro (version 12, jmp.com) and GraphPad PRISM (version 8.0.0 for Windows, www.graphpad.com). The data were checked for normality using Skewness-Kurtosis and Goodness of Fit. If normal, parametric methods were applied to calculate the significance between different experimental groups at single time point (p < 0.05 were considered significant). When groups had an equal n-number, two-way ANOVA was used (immunohistological data); when n-number differed between the groups, one-way ANOVA was utilized instead (behavior data). If significant, post-hoc analysis was applied using Turkey’s multiple comparison test/Honest Significant Difference (HSD). The Turkey’s test compares all possible pairs of means between different treatment groups and is considered significant if p < 0.05. The Shapiro–Wilk test was used if data were not normally distributed. Repeated-measure analysis of variance (MANOVA) was used to analyze continuous performance of mice in the Barnes Maze and Rotarod (p < 0.05 is significant). Error bars represent the standard error of the mean.

### Compliance statement

The study was carried out in compliance with the ARRIVE guidelines.

## Results

### Motor assessment

**Figure 3:**
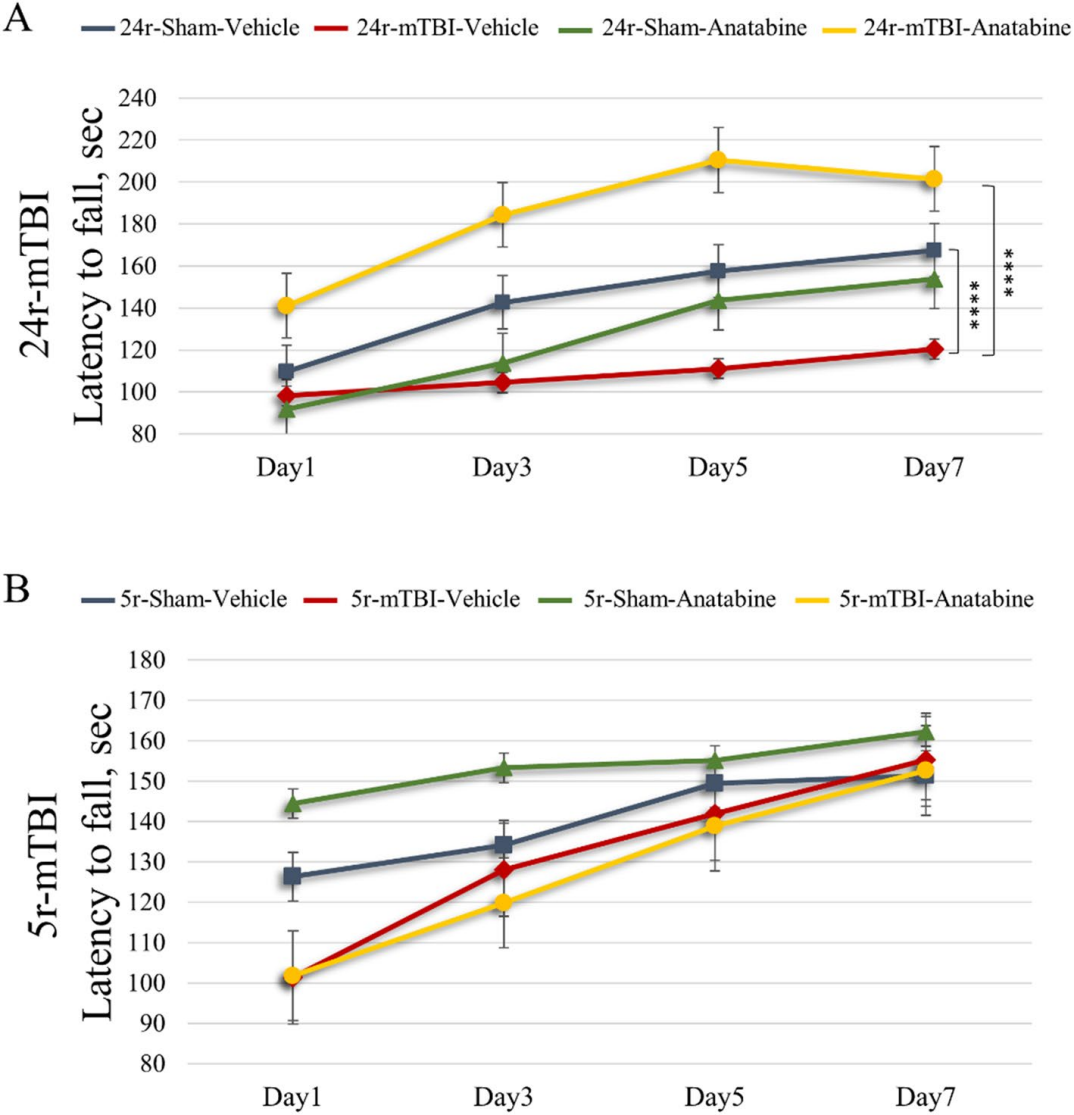
Evaluation of the effect of TBI and of anatabine on motor performance. (**A**) 24r-mTBI-vehicle mice exhibited a decrease in latency over the 7-day period compared to 24r-Sham-Vehicle controls (p < 0.0001). Treatment with anatabine in 24r-mTBI mice increased latency from Day1 to Day7 (p < 0.0001). (**B**) 5r-mTBI-Vehicle mice exhibited a decreased latency compared to 5r-Sham-Vehicle on Day1 and Day 3 but not on Day5 and Day7. Mice of 5r-mTBI-Anatabine group had no difference in latency compared to 5r-mTBI-Vehicle mice (p > 0.05). All data were normalized to baseline and analyzed using MANOVA.

For the 24r-mTBI mice, rotarod assessment showed a TBI-induced 37.5% decrease in sensorimotor performance of the 24r-mTBI-vehicle compared to the 24r-sham-vehicle group (Fig. 3A). The 24r-mTBI-vehicle mice had a lower latency to fall compared to their controls during a 7-day trial (Fig. 3A; 24r-mTBI-vehicle vs 24r-sham-vehicle: 120 ± 3 s vs 168 ± 17 s; p < 0.0001, MANOVA). Treatment with anatabine in the 24r-mTBI mice significantly increased the latency to fall compared to vehicle treated 24r-mTBI mice (Fig. 3A; 24r-mTBI-anatabine vs 24r-mTBI-vehicle: 206 ± 23 s vs 120 ± 3 s; p < 0.0001, MANOVA).

In the 5r-mTBI mice, a decreased latency to fall was observed in 5r-mTBI-vehicle vs 5r-sham-vehicle mice on days 1 and 3 but not on days 5 and 7, and no overall difference was observed throughout 7 days as measured by MANOVA (Fig. 3B; p > 0.05, MANOVA). 5r-mTBI-vehicle mice showed a motor recovery behavior over the duration of the testing. No difference was observed between the 5r-mTBI-vehicle and 5r-mTBI-anatabine mice throughout the 7-day trial.

### Memory assessment

#### 24r-mTBI

**Figure 4:**
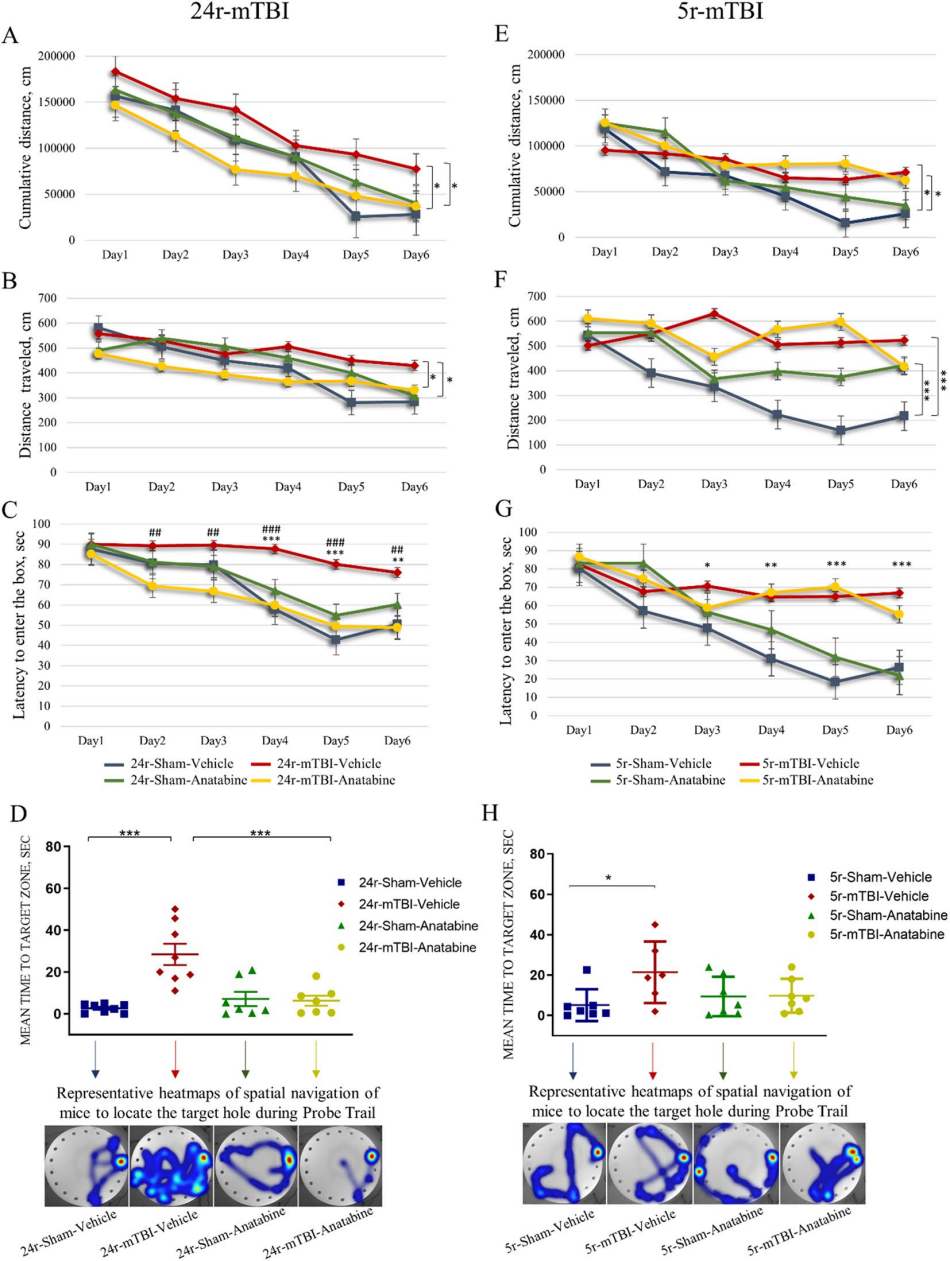
Evaluation of learning and memory in 24r-mTBI and 5r-mTBI mice. Anatabine treatment improved spatial memory deficits in the 24r-mTBI but not 5r-mTBI mice. In the 24r-mTBI cohort, 24r-mTBI-vehicle mice exhibited longer cumulative distance ((**A**); p < 0.05), distance traveled ((**B**); p < 0.05), and latency to enter the box on days 4–6 (**C**) compared to 24r-sham vehicle during the acquisition trial. Similarly, in the 5r-mTBI cohort, 5r-mTBI-vehicle mice exhibited longer cumulative distance ((**E**); p < 0.05), distance traveled ((**F**); p < 0.001), and latency to enter the box on days 3–6 (**G**) compared to 5r-sham vehicle. 24r-mTBI-anatabine mice showed a decrease in cumulative distance ((**A**); p < 0.05), distance traveled ((**B**); p < 0.05) and latency (**C**) compared to 24r-mTBI- vehicle mice. Anatabine did not attenuate memory deficits in the 5r-mTBI-anatabine group during the acquisition trial. During the probe trial, 24r-mTBI increased mean time to target zone compared to sham-vehicle mice ((**D**); p < 0.001), which was decreased in the 24r-mTBI-anatabine mice vs 24r-mTBI ((**D**); p < 0.001). In the 5r-mTBI group, 5r-mTBI-vehicle mice had an increase in mean time compared to 5r-sham-vehicle ((**H**); p < 0.05) but no anatabine induced decrease was detected in the 5r-mTBI-anatabine mice, however the trend is present. Latency in 5r-mTBI-anatabine mice was not significantly different from the 5r-sham-vehicle mice (p = 0.082). Statistical significance for the cumulative distance and distance traveled were analyzed using MANOVA; the mean time to target hole was analyzed using one-way ANOVA.

Over the 6 days, all groups showed an improved performance as measured by cumulative distance, distance traveled and latency to enter the box. 24r-mTBI-vehicle mice showed a 68% increase in the cumulative distance traveled compared to the 24r-sham-vehicle by the end of a 6-day acquisition trial (Fig. 4A; p < 0.05, MANOVA). For distance traveled, 24r-mTBI-vehicle mice showed a 33% increase compared to 24r-sham-vehicle mice (Fig. 4B; p < 0.05, MANOVA). In the 24r-mTBI-vehicle mice, the latency to enter the box was 43% increased compared to 24r-sham-vehicle (Fig. 4C; day 6 p < 0.01, one-way ANOVA). Latency was not different between 24r-sham-vehicle and 24r-mTBI-vehicle until day 4 (day 4 p < 0.001, day 5 p < 0.001, day 6 p < 0.01, one-way ANOVA). 24r-mTBI-anatabine mice had a 65% decrease in cumulative distance compared to 24r-mTBI-vehicle group (p < 0.05, MANOVA). For distance traveled, 24r-mTBI-anatabine mice exhibited a 30% decrease compared to 24r-mTBI-vehicle. Treatment with anatabine significantly reduced latency to enter the box in the injured mice starting on day 2 of the acquisition trial (24r-mTBI-anatabine vs 24r-mTBI-vehicle: day 2 p < 0.01, day 3 p < 0.01, day 4 p < 0.001, day 5 p < 0.01, day 6 p < 0.01, one-way ANOVA). During the probe trial, 24r-mTBI-vehicle mice spent 90% longer to locate the target hole compared to the 24r-sham-vehicle (Fig. 4D; 24r-mTBI-vehicle vs 24r-sham-vehicle: 28.3 ± 21 s vs 2.6 ± 1.2 s; p < 0.05, one-way ANOVA). In the 24r-mTBI-anatabine mice, mean time was 82.3% decreased compared to 24r-mTBI-vehicle (24r-mTBI-anatabine vs 24r-mTBI-vehicle: 4.9 ± 4.2 s vs 28.3 ± 21 s; p < 0.05, one-way ANOVA).

#### 5r-mTBI

The acquisition trial showed an overall decrease in the cumulative distance, distance traveled, and latency to enter the target box for all 5r-mTBI experimental groups over the 6-day period. 5r-mTBI-vehicle mice showed a 67% increase in the cumulative distance compared to the 5r-sham-vehicle by the end of a 6-day acquisition trial (Fig. 4E; p < 0.05, MANOVA). Similarly, in the 5r-mTBI-vehicle mice, distance traveled was 52% increased (Fig. 4F; p < 0.001, MANOVA) and latency to enter the box was 57.8% increased, compared to 5r-sham-vehicle (Fig. 4G; day 6 p < 0.001, one-way ANOVA). Latency to enter the box was increased in the 5r-mTBI-vehicle mice compared to 5r-sham-vehicle starting day 3 after the initiation of the trial (days 3 p < 0.05, day 4 p < 0.01, day 5–6 p < 0.001, one-way ANOVA). No anatabine-induced improvements were shown for any of these parameters in the 5r-mTBI group (5r-mTBI-anatabine vs 5r-mTBI-vehicle: cumulative distance p > 0.05, distance traveled p > 0.05, latency p > 0.05). In the probe trial, latency to locate the target hole was increased in the 5r-mTBI-vehicle mice vs 5r-sham-vehicle (Fig. 4H; p < 0.05, one-way ANOVA) but no anatabine-induced decrease in the 5r-TBI mice was shown compared to 5r-mTBI-vehicle (Fig. 4H; p > 0.05, one-way ANOVA).

There was a greater variation in the signal in 5r-mTBI-vehicle vs 5r-sham-vehicle mice compared to 24r-mTBI-vehicle vs 24r-sham-vehicle. In both cohorts, TBI-anatabine groups were not significantly different from their respective shams.

### Disinhibition assessment

**Figure 5:**
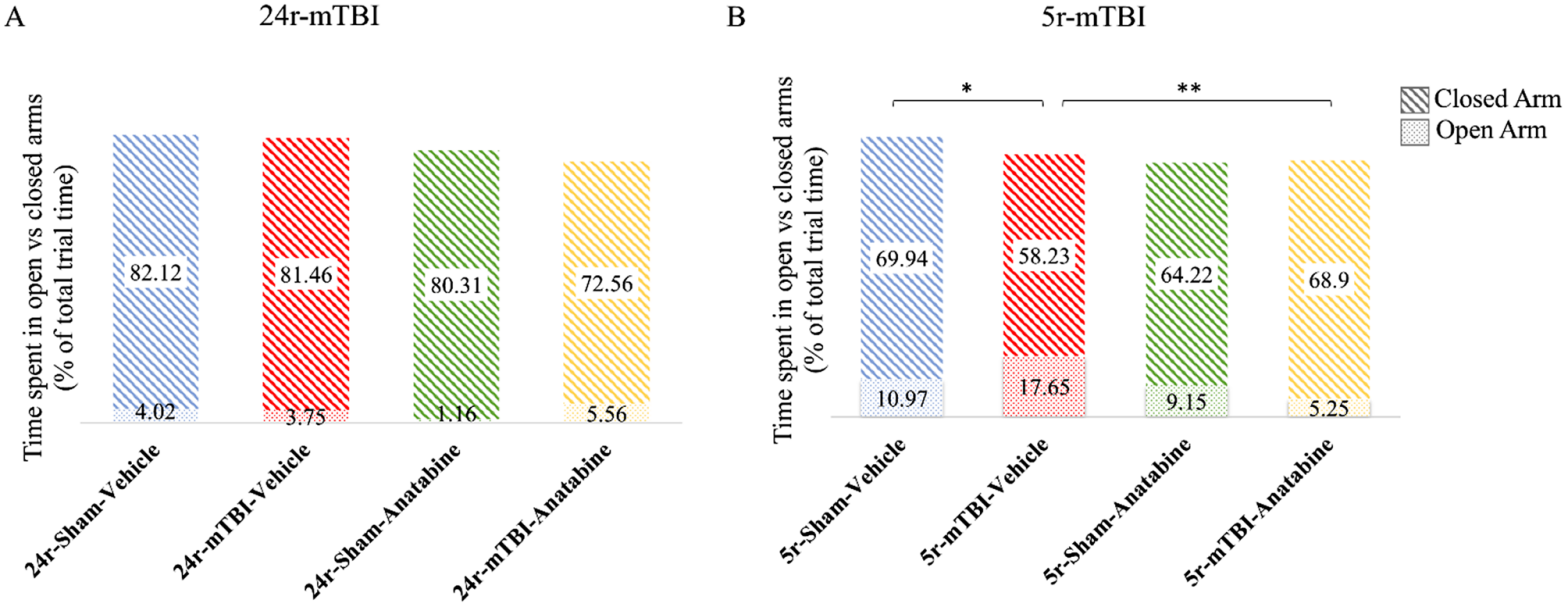
Evaluation of disinhibition (time spent in open vs closed arms) in the 24r-mTBI and 5r-mTBI mice using the EPM. (**A**) There was no difference in time spent in open vs closed arms between the 24r-mTBI-vehicle (3.75 vs 81.46) and 24r-sham-vehicle (4.02 vs 82.12) and between the 24r-mTBI-vehicle (3.75 vs 81.46) and 24r-mTBI-anatabine (5.56 vs 72.56). (**B**) There was an increase in time spent in the open arms in 5r-mTBI-vehicle vs 5r-mTBI-sham mice (17.65 vs 10.97, p < 0.05). In the 5r-mTBI-anatabine mice, time spent in the open arms decreased compared to 5r-mTBI-vehicle (5.25 vs 17.65, p < 0.01). Statistical significance was analyzed using one-way ANOVA.

EPM analysis showed a TBI effect on disinhibition behavior in the 5r-mTBI but not 24r-mTBI mice. In the 5r-mTBI group, 5r-mTBI-vehicle mice spent more time in the open arm compared to the 5r-sham-vehicle (Fig. 5B; p < 0.05, one-way ANOVA). Treatment with anatabine reduced time spent in the open arm in the 5r-mTBI mice vs 5r-mTBI-Vehicle controls (Fig. 5B; p < 0.01, one-way ANOVA). In the 24r-mTBI mice, no differences were observed between vehicle and anatabine treated mice (Fig. 5A).

### Immunohistochemistry

**Figure 6:**
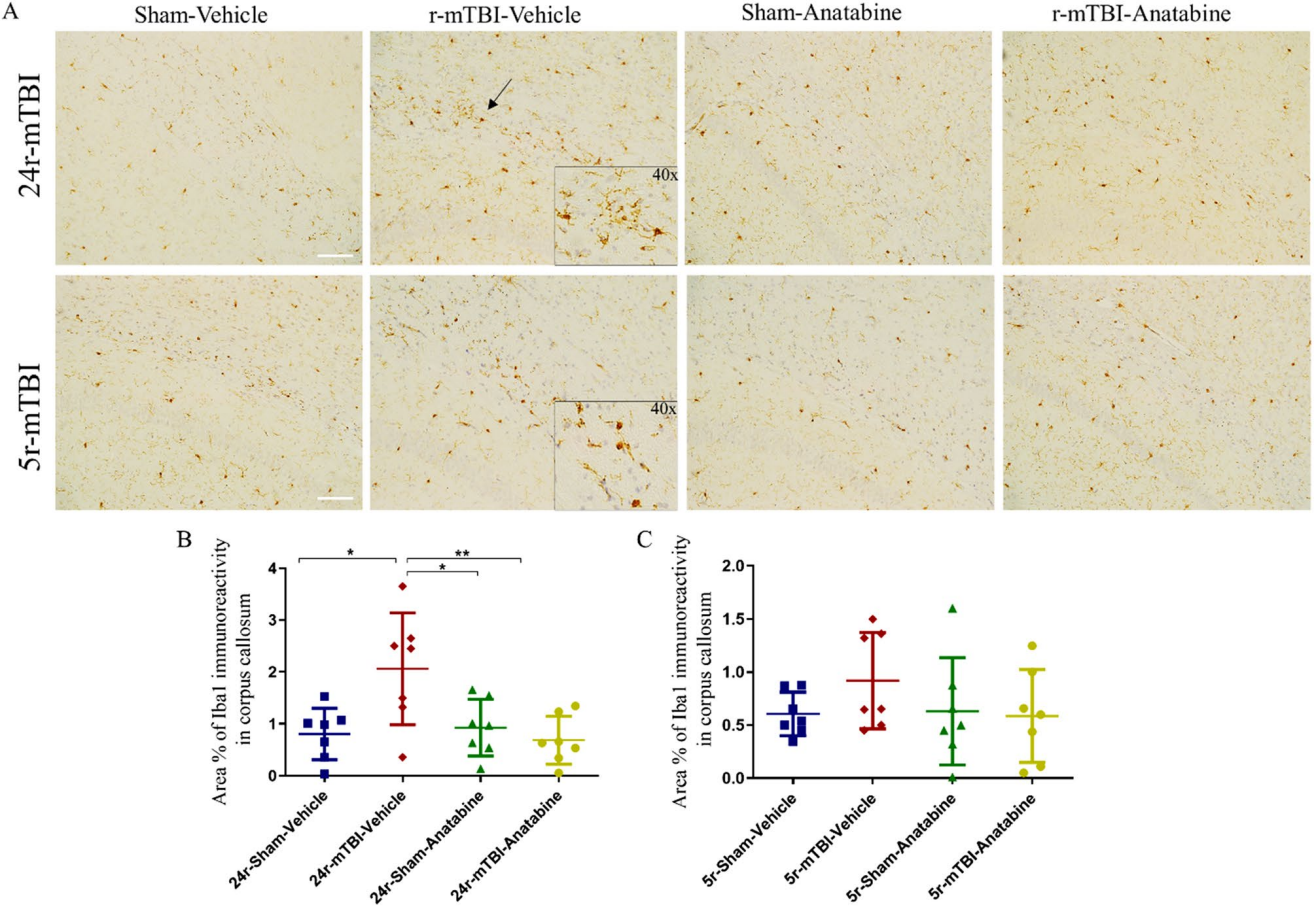
Evaluation of the effect of anatabine on microglia. (**A**) Representative images of Iba1 in corpus callosum in the 5r-mTBI and 24r-mTBI mice. Arrow indicates immunoreactive microglia. (**B**) 24r-mTBI-vehicle mice had an increase in Iba1 signal compared to 24r-sham-vehicle (p < 0.05). (**C**) In 5r-mTBI-vehicle mice, no increased Iba1 was detected. Anatabine decreased Iba1 in 24r-mTBI mice (24r-mTBI-anatabine vs 24r-mTBI-vehicle p < 0.05) but not in 5r-mTBI. Inserts of 40× images of reactive microglia are included in pictures of each r-mTBI-vehicle group. Data analyzed using two-way ANOVA. Scale bars equal 20 µm.

In the 24r-mTBI but not 5r-mTBI mice, Iba1 signal was increased in the corpus callosum in the injured mice compared to their respective controls (Fig. 6; 5r-mTBI-vehicle vs 5r-sham-vehicle p > 0.05, 24r-mTBI-vehicle vs 24r-sham-vehicle p < 0.05, one-way ANOVA). Compared to 24r-mTBI-vehicle mice, 24r-mTBI-anatabine mice showed attenuated microglia activation that was close to 24r-sham-vehicle levels (Fig. 6B,C; 24r-mTBI-anatabine vs 24r-mTBI-vehicle p < 0.05, 24r-mTBI-anatabine vs 24r-sham-vehicle p > 0.05, one-way ANOVA).

**Figure 7:**
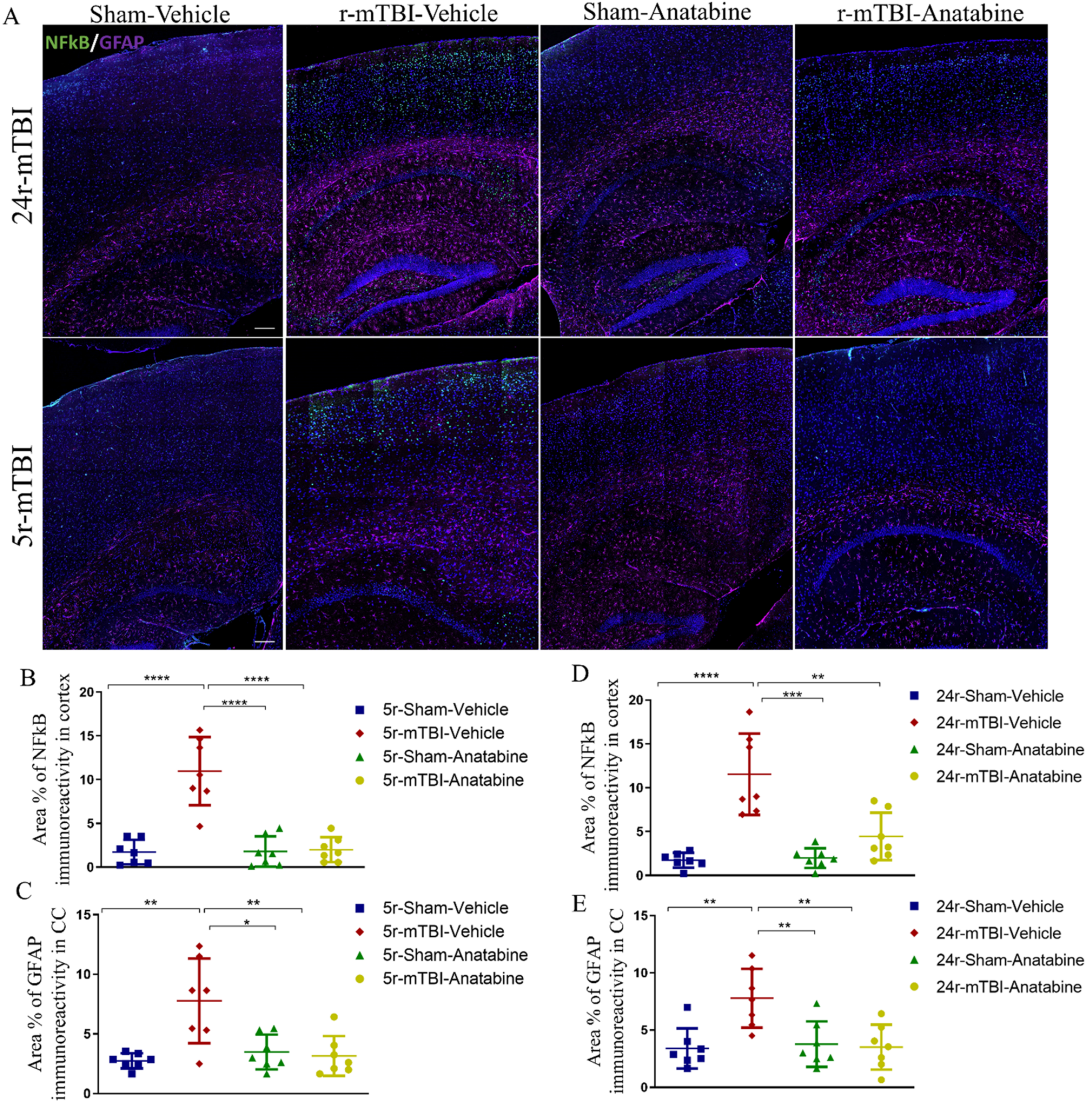
Immunofluorescent analysis of GFAP and NFkB. (**A**) Representative images of NFkB/GFAP in cortex, corpus callosum (CC) and hippocampus. (**B**,**C**) Quantification of area coverage of NFkB in cortex and GFAP in CC, respectively, in 5r-mTBI cohort. (**D**,**E**) quantification of area coverage of NFkB in cortex and GFAP in CC, respectively, in 24r-mTBI cohort. In the 24r-mTBI group, TBI produced a significant increase of GFAP in CC ((**E**), p < 0.001) and of NFkB in cortex ((**D**), p < 0.0001) compared to 24r-sham-vehicle. Similarly, in the 5r-mTBI group, TBI induced GFAP ((**C**), p < 0.001) and NFkB ((**B**), p < 0.0001) immunoreactivity compared to 5r-sham-vehicle mice. Treatment with anatabine decreased GFAP (24r-mTBI p < 0.01; 5r-mTBI p < 0.01) and NFkB (24r-mTBI p < 0.001; 5r-mTBI p < 0.0001) in r-mTBI mice compared to the respective r-mTBI-vehicle mice. Data analyzed using two-way ANOVA. Scale bar equal 200 µm.

Immunofluorescent assessment for the astroglial marker GFAP revealed an increase in astrogliosis in the injured mice compared to the sham for both models (Fig. 7A). In both 5r-mTBI and 24r-mTBI groups, an increased area of GFAP immunoreactivity was observed in the CA1 region and in the body of corpus callosum but not in the cortex. Quantitative analysis showed an increase in the area % of GFAP cells in the corpus callosum of the r-mTBI mice compared to sham-vehicle mice (Fig. 7D: 5r-mTBI-vehicle vs 5r-sham-vehicle p < 0.001; Fig. 7B: 24r-mTBI-vehicle vs 24r-sham-vehicle p < 0.001). After treatment with anatabine, both 5r-mTBI and 24r-mTBI showed a reduction in GFAP immunoreactivity in the corpus callosum (5r-mTBI-anatabine vs 5r-mTBI-vehicle p < 0.01; 24r-mTBI-anatabine vs 24r-mTBI-vehicle p < 0.01, one-way ANOVA). Immunoreactivity for NFκB was detected in the cortex and hippocampal areas CA1, CA3, and DG. In the cortex, quantitative analysis for NFκB revealed an increase in both cohorts in the r-mTBI groups compared to their respective controls: 5r-mTBI (Fig. 7E: 5r-mTBI-vehicle vs 5r-sham-vehicle p < 0.0001, one-way ANOVA) and 24r-mTBI (Fig. 7C: 24r-mTBI-vehicle vs 24r-sham-vehicle p < 0.0001, one-way ANOVA). Images at higher magnification (× 20) revealed a NFκB /DAPI colocalization in the r-mTBI mice in the CA1 area of hippocampus and throughout cortex (Supplementary Fig. S1). Their increase was attenuated after treatment with anatabine in all areas (quantitative analysis for cortex: 5r-mTBI-anatabine vs 5r-mTBI-vehicle p < 0.0001; 24r-mTBI-anatabine vs 24r-mTBI-vehicle p < 0.001).

**Figure 8:**
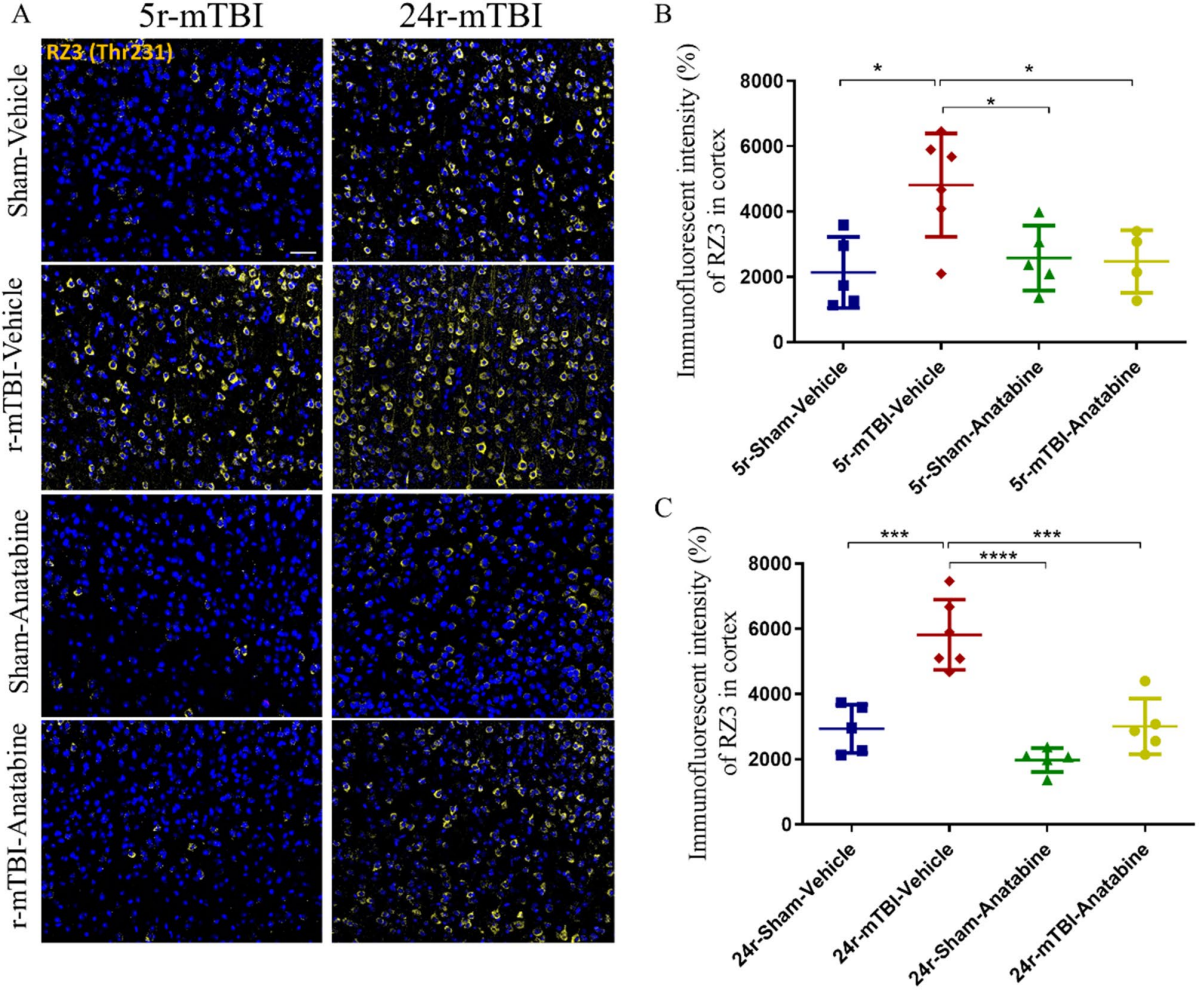
Evaluation of the effect of anatabine on RZ3 (Thr231) in cortex. (**A**) Representative images of RZ3 (Thr231) in cortex. TBI induced an increase of RZ3 (Thr231) in both 5r-mTBI ((**B**), p < 0.05) and 24r-mTBI ((**C**), p < 0.001) mice compared to the respective sham-vehicle. In anatabine treated mice, RZ3 (Thr231) was decreased in both 5r-mTBI (p < 0.05) and 24r-mTBI (p < 0.001) mice compared to the respective r-mTBI-vehicle. Data analyzed using two-way ANOVA. Scale bars equal 20 µm.

Immunofluorescent analysis for phosphorylated tau showed a significant increase of RZ3 (Thr231) signal in the cortex (Fig. 8A) of both 5r-mTBI and 24r-mTBI mice compared to their respective sham-vehicle mice (Fig. 8B: 5r-mTBI-vehicle vs 5r-sham-vehicle: p < 0.05; Fig. 8C: 24r-mTBI-vehicle vs 24r-sham-vehicle p < 0.001, one-way ANOVA). Anatabine treatment decreased RZ3 (Thr231) fluorescent intensity in the 5r-mTBI (vs 5r-mTBI-vehicle p < 0.05, one-way ANOVA) and 24r-mTBI (vs 24r-mTBI-vehicle p < 0.001, one-way ANOVA).

## Discussion

This study focused on delayed treatment with anatabine in hTau mice in two different models of r-mTBI. The selection of hTau mice was driven by our attempt to mimic chronic tau phosphorylation in human r-mTBI while the selection of two injury paradigms was aimed to represent the diversity of clinical r-mTBI in regard to frequency and number of injuries. Both our models induced cognitive deficits as well as strong glial response and tau phosphorylation at 6 months post-last mTBI. We further found that anatabine decreased GFAP, NFκB and p-tau in both models, however its effect on behavior was mostly demonstrated in the 24r-mTBI model, but not in the 5r-mTBI mice.

Both 5-rmTBI and 24r-mTBI mice (vehicle treated), despite age difference at the time of testing, exhibited strong injury-dependent astrogliosis in the corpus callosum and hippocampus 6-months post last mTBI. No reactive astrocytes were observed in cortex within the injury site, consistent with our previous findings showing that this pathology is found only during the acute phase in our models^18,20,30^. Such temporal alterations probably indicate that the astrogliosis induced in the cortex shortly after TBI is associated with mechanical deformation of the tissue^31^, and it resolves after the termination of the mechanical stress. On the contrary, white matter has been shown to be more vulnerable to TBI leading to long-lasting pathology^32^. White matter is comprised of interconnected axon tracts that are especially susceptible to stretch forces at the grey-white matter junctions. In fact, most axonal injury in white matter is caused not by shear and stress forces but as a result of secondary injury events such as white matter atrophy and demyelination^33^. White matter damage has been recorded in mTBI animal models at > 1 year post injury^19,20,23,25,34^ and in TBI patients^6^, and has been proposed to be linked with cognitive deficits^35–37^. We also observed an increase in the transcription factor NFκB p-65, a critical regulator of immune response and inflammation, in both our models at 6 months post-injury. Under baseline conditions, NFκB is found in the cytosol, but upon activation a p65/p50 heterodimer translocates to the nucleus where it binds specific DNA sequences and promotes the expression of inflammatory cytokines^38^. Our confocal images of samples co-stained with NFκB p-65 and DAPI showed nuclear localization of NFκB in the hippocampus of r-mTBI mice. Moreover, both models of r-mTBI exhibited tau phosphorylation in the cortex and the CA1 area of the hippocampus at 6 months post injury. We have previously shown an increase in total and p-tau (Thr 231) in 24r-mTBI hTau mice at 3 months post-injury^23^ but these data represent our first time reporting a similarly persistent p-tau signal in the 5r-mTBI hTau mice.

Treatment with anatabine for 3 months after a delay of 3 months post-last injury attenuated TBI-induced pathology in both models of r-mTBI. We observed a decrease of GFAP, NFκB and p-tau, markers that were increased in response to TBI but not in sham mice. These data are supported by our previous findings where at 9 months post 5r-mTBI, anatabine reduced inflammation and improved cognitive functions in wild-type mice^16^. Interestingly, in our previous study, we observed that despite the positive effects of an initial 9 months of anatabine treatment, a subsequent 9-month cessation of anatabine resulted in a return of the injury-dependent Iba1 signal indicating that microgliosis was suppressed but not completely removed by anatabine. Thus, it appears that treatment with anatabine does not target the underlying causes of inflammation but exhibits a modulating effect for the period of treatment. However, in that same study, delayed treatment with anatabine, beginning at 9 months post-last injury, successfully reduced Iba1 to sham levels at the 18-month time point, prompting our current study design. Therapeutic responses to anatabine in our current study suggest that p-tau pathology may represent a downstream consequence of inflammation mediated through the chronic upregulation of NFκB. There have been a few studies showing that tau phosphorylation is regulated through the early growth response-1 (EGR1) transcriptional factor, which is one of the gene targets of NFκB^39–41^. A possible NFκB—p-tau signaling may represent a potential target in r-mTBI, although additional research is required.

At 6 months post-last mTBI, both models exhibited injury-induced memory and learning deficits whereas, unexpectedly, only the 5r-mTBI, but not the 24r-mTBI mice, showed disinhibition behavior. Increased disinhibition is an indicator of risk-taking behavior, which, in humans, is linked with hyperactivity and aggression. A long-lasting disinhibition (time spent in the open arm) was found in other models of 5-hit mTBI in WT mice^42^ but no study has addressed the effect of r-mTBI over several months, such as our 24r-mTBI model, on such behavior. The lack of disinhibitory behavior in the 24r-mTBI, a more severe model than 5r-mTBI, might be linked to a 3-months age difference between the models at the time of testing. Disinhibition may be triggered by natural aging in hTau mice and mask the TBI specific response in our 24r-mTBI model.

The effect of anatabine on motor and cognitive outcomes showed inconsistent results between the two models. In the 24r-mTBI mice, anatabine mitigated both sensorimotor and memory impairments, whereas in the 5r-mTBI group, anatabine did not exhibit therapeutic effect on the same parameters. So far, we are unable to elaborate on the diverse effect of anatabine on cognitive improvements, especially when ameliorated pathology was shown in both models. It appears that the behavioral effects of anatabine is stronger or, at least, is easier to discriminate if the injury is more severe. A possible explanation may be that anatabine acts through the alternative mechanisms which differ between the models due to the final age of mice and the injury model. Expression of the primary target of anatabine, the α7 subunit of nAChR, is known to fluctuate with age and may be differently expressed in 24r-mTBI mice, which were 80 days older than the 5r-mTBI mice. The α7 nAChR is also involved in the regulation of calcium signaling which is a key feature of secondary injury involved in cellular apoptosis and oxidative stress and may represent an alternative target for anatabine^43^. Moreover, α7 is not only expressed by neurons but also by microglia, which showed a different degree of response between the two models which could potentially alter α7 expression and, consequently, the anatabine effect. Another possible reason for diverse responses to anatabine between the two models might derive from significant age difference at the time of treatment and behavior testing. Both cohorts were exposed to injuries at 3 months of age but, due to a longer paradigm of 24-rmTBI, their age at treatment varied by 3 months. Hence, in 24r-mTBI mice, more distinguished cognitive deficits can be driven not only by r-mTBI but also by aging, a trend that is less evident in 5r-mTBI mice.

A lack of depth and detail in preclinical studies has been cited as a leading reason for the failure of clinical translation for TBI^44^. Factors hindering successful translation of preclinical findings to the clinic may include inadequate design of injury procedures, inappropriate timing of treatment and inability to reproduce the findings across different animal models prior to moving into the more diverse populations inherent in human trials. The largest study designed to address these nuances and to compare the efficacy of potential treatments across different injury models in order to enhance the translation of therapies from rodents to men is the Operation Brain Trauma Therapy (OBTT) consortium^45^. This multi-center consortium has tested 5 therapies in rat models of fluid percussion injury (FPI), controlled cortical impact (CCI) and penetrating ballistic-like brain injury (PBBI). Interestingly, 4 of them underperformed relative to initial studies in individual models of TBI and only one (levetiracetam) demonstrated partial efficacy across different models^46^. Overall, this study demonstrated that the most promising preclinical therapeutic candidates fail to reproduce similar results across various models. As counter to this conclusion, the OBTT approach has several limitations that may undermine its translational potential. First, the TBI models used represent very different mechanisms with diffuse (FPI) and focal (CCI and PBBI) injuries, each of them causing distinct pathology. Second, all treatments were administered at very acute time points ranging from 15 min to 24 h post-TBI. This time window may have missed treatments that require chronic administration relevant for cases of human mild and sometimes even moderate TBI.

We would like to acknowledge that our study also has limitations, and care should be taken in comparing between two models of r-mTBI. The age of mice at the time of treatment and euthanasia was different between the cohorts. Due to the longer duration of 24r-mTBI paradigm (12 weeks) than 5r-mTBI (9 days), mice that entered the study at 3 months of age, were older in the 24r-mTBI group (12 months) than 5r-mTBI (9 months) by the end of the study. The age difference at each step (i.e. end of injury, beginning of treatment, behavior assessment, euthanasia, and tissue collection) might have contributed to the variations of treatment outcomes as discussed above. For example, aging may exacerbate TBI-induced cognitive deficits leading to a more prominent response. The best way to eliminate this “age background” is by using sham mice for both vehicle and anatabine groups, as have been done in this study. However, we would also like to highlight that matching our cohorts by the age at which treatment administration began, while retaining our desired 3-month treatment delay, would have resulted in age differences at the time of injury which we considered would have greater impact on comparison between the models. Therefore, our primary goal was to match the ages of mice at the time of injury. Nevertheless, careful interpretation of the results and further work are crucial.

## Conclusion

Our study highlights a delayed treatment paradigm in two chronic models of r-mTBI as an appropriate strategy for testing anti-inflammatory therapeutics. Despite age difference at the time of treatment, delayed administration of anatabine inhibited inflammation and reduced tau phosphorylation underlying a possible link between the two in chronic r-mTBI pathology. We also demonstrated therapeutic variability between different models of r-mTBI in hTau mice, which underscores the necessity to test treatments across multiple models. To better investigate heterogeneity of different models and treatment paradigms, and to identify therapeutic targets common between all our models, we plan next to conduct a proteomic and phosphoproteomic analyses of these brain tissue samples. Identification of molecular level TBI- and/or treatment-dependent responses will potentially reveal targets for further investigation as critical determinants and possible foci for intervention in TBI pathobiology.

## Supplementary Information

Supplementary Figure S1.

## References

[1] Cassidy JD, Carroll LJ, Peloso PM, Borg J, von Holst H, Holm L, Kraus J, Coronado VG (2004). Incidence, risk factors and prevention of mild traumatic brain injury: Results of the WHO Collaborating Centre Task Force on Mild Traumatic Brain Injury. J. Rehabil. Med..

[2] Maas AIR, Menon DK, Adelson PD, Andelic N, Bell MJ, Belli A, Bragge P, Zumbo F (2017). Traumatic brain injury: Integrated approaches to improve prevention, clinical care, and research. Lancet Neurol..

[3] Gardner RC, Yaffe K (2015). Epidemiology of mild traumatic brain injury and neurodegenerative disease. Mol. Cell. Neurosci..

[4] Kiernan PT, Montenigro PH, Solomon TM, McKee AC (2015). Chronic traumatic encephalopathy: A neurodegenerative consequence of repetitive traumatic brain injury. Semin. Neurol..

[5] Gentleman SM, Leclercq PD, Moyes L, Graham DI, Smith C, Griffin WST, Nicoll JAR (2004). Long-term intracerebral inflammatory response after traumatic brain injury. Forens. Sci. Int..

[6] Johnson VE, Stewart JE, Begbie FD, Trojanowski JQ, Smith DH, Stewart W (2013). Inflammation and white matter degeneration persist for years after a single traumatic brain injury. Brain.

[7] McKee AC, Cairns NJ, Dickson DW, Folkerth RD, Dirk KC, Litvan I, Perl DP, Stein TD, Vonsattel JP, Stewart W, Tripodis Y, Crary JF, Bieniek KF, Dams-O’Connor K, Alvarez VE, Gordon WA (2016). The first NINDS/NIBIB consensus meeting to define neuropathological criteria for the diagnosis of chronic traumatic encephalopathy. Acta Neuropathol..

[8] Bergold PJ (2016). Treatment of traumatic brain injury with anti-inflammatory drugs. Exp. Neurol..

[9] Meythaler J, Fath J, Fuerst D, Zokary H, Freese K, Martin HB, Reineke J, Peduzzi-Nelson J, Roskos PT (2019). Safety and feasibility of minocycline in treatment of acute traumatic brain injury. Brain Inj..

[10] Robertson CS, McCarthy JJ, Miller ER, Levin H, McCauley SR, Swank PR (2017). Phase II clinical trial of atorvastatin in mild traumatic brain injury. J. Neurotrauma.

[11] Stein DG (2015). Embracing failure: What the phase III progesterone studies can teach about TBI clinical trials. Brain Inj..

[12] Gold EM, Su D, López-Velázquez L, Haus DL, Perez H, Lacuesta GA, Anderson AJ, Cummings BJ (2013). Functional assessment of long-term deficits in rodent models of traumatic brain injury. Regen. Med..

[13] Verma M, Beaulieu-Abdelahad D, Ait-Ghezala G, Li R, Crawford F, Mullan M, Paris D (2015). Chronic anatabine treatment reduces Alzheimer’s disease (AD)-like pathology and improves socio-behavioral deficits in a transgenic mouse model of AD. PLoS ONE.

[14] Paris D, Beaulieu-Abdelahad D, Abdullah L, Bachmeier C, Ait-Ghezala G, Reed J, Verma M, Crawford F, Mullan M (2013). Anti-inflammatory activity of anatabine via inhibition of STAT3 phosphorylation. Eur. J. Pharmacol..

[15] Paris D, Beaulieu-Abdelahad D, Mullan M, Ait-Ghezala G, Mathura V, Bachmeier C, Crawford F, Mullan MJ (2013). Amelioration of experimental autoimmune encephalomyelitis by Anatabine. PLoS ONE.

[16] Ferguson S, Mouzon B, Paris D, Aponte D, Abdullah L, Stewart W, Mullan M, Crawford F (2016). Acute or delayed treatment with anatabine improves spatial memory and reduces pathological sequelae at late time-points after repetitive mild traumatic brain injury. J. Neurotrauma.

[17] Levin ED, Hao I, Burke DA, Cauley M, Hall BJ, Rezvani AH (2014). Effects of tobacco smoke constituents, anabasine and anatabine, on memory and attention in female rats. J. Psychopharmacol..

[18] Mouzon B, Chaytow H, Crynen G, Bachmeier C, Stewart J, Mullan M, Stewart W, Crawford F (2012). Repetitive mild traumatic brain injury in a mouse model produces learning and memory deficits accompanied by histological changes. J. Neurotrauma.

[19] Mouzon BC, Bachmeier C, Ferro A, Ojo JO, Crynen G, Acker CM, Davies P, Mullan M, Stewart W, Crawford F (2014). Chronic neuropathological and neurobehavioral changes in a repetitive mild traumatic brain injury model. Ann. Neurol..

[20] Mouzon BC, Bachmeier C, Ojo JO, Acker CM, Ferguson S, Paris D, Ait-Ghezala G, Crynen G, Davies P, Mullan M, Stewart W, Crawford F (2018). Lifelong behavioral and neuropathological consequences of repetitive mild traumatic brain injury. Ann. Clin. Transl. Neurol..

[21] McKee AC, Daneshvar DH, Alvarez VE, Stein TD (2014). The neuropathology of sport. Acta Neuropathol..

[22] Andorfer C, Kress Y, Espinoza M, De Silva R, Tucker KL, Barde YA, Duff K, Davies P (2003). Hyperphosphorylation and aggregation of tau in mice expressing normal human tau isoforms. J. Neurochem..

[23] Ojo JO, Mouzon B, Algamal M, Leary P, Lynch C, Abdullah L, Evans J, Mullan M, Bachmeier C, Stewart W, Crawford F (2016). Chronic repetitive mild traumatic brain injury results in reduced cerebral blood flow, axonal injury, gliosis, and increased T-tau and tau oligomers. J. Neuropathol. Exp. Neurol..

[24] Ojo JO, Mouzon B, Greenberg MB, Bachmeier C, Mullan M, Crawford F (2013). Repetitive mild traumatic brain injury augments tau pathology and glial activation in aged hTau mice. J. Neuropathol. Exp. Neurol..

[25] Mouzon B, Bachmeier C, Ojo J, Acker C, Ferguson S, Crynen G, Davies P, Mullan M, Stewart W, Crawford F (2019). Chronic white matter degeneration, but no tau pathology at one-year post-repetitive mild traumatic brain injury in a tau transgenic model. J. Neurotrauma.

[26] Ojo JO, Bachmeier C, Mouzon BC, Tzekov R, Mullan M, Davies H, Stewart MG, Crawford F (2015). Ultrastructural changes in the white and gray matter of mice at Chronic time points after repeated concussive head injury. J. Neuropathol. Exp. Neurol..

[27] Tzekov R, Quezada A, Gautier M, Biggins D, Frances C, Mouzon B, Jamison J, Mullan M, Crawford F (2014). Repetitive mild traumatic brain injury causes optic nerve and retinal damage in a mouse model. J. Neuropathol. Exp. Neurol..

[28] Tzekov R, Dawson C, Orlando M, Mouzon B, Reed J, Evans J, Crynen G, Mullan M, Crawford F (2016). Sub-Chronic neuropathological and biochemical changes in mouse visual system after repetitive mild traumatic brain injury. PLoS ONE.

[29] Bachmanov AA, Reed DR, Beauchamp GK, Tordoff MG (2002). Food intake, water intake, and drinking spout side preference of 28 mouse strains. Behav. Genet..

[30] Ferguson SA, Mouzon BC, Lynch C, Lungmus C, Morin A, Crynen G, Carper B, Bieler G, Mufson EJ, Stewart W, Mullan M, Crawford F (2017). Negative impact of female sex on outcomes from repetitive mild traumatic brain injury in hTau mice is age dependent: A chronic effects of Neurotrauma Consortium study. Front. Aging Neurosci..

[31] Burda JE, Bernstein AM, Sofroniew MV (2016). Astrocyte roles in traumatic brain injury. Exp. Neurol..

[32] Braun M, Vaibhav K, Saad NM, Fatima S, Vender JR, Baban B, Hoda MN, Dhandapani KM (2017). White matter damage after traumatic brain injury: A role for damage associated molecular patterns. Biochim. Biophys. Acta.

[33] Büki A, Povlishock JT (2006). All roads lead to disconnection? Traumatic axonal injury revisited. Acta Neurochir..

[34] Bramlett HM, Dietrich WD (2002). Quantitative structural changes in white and gray matter 1 year following traumatic brain injury in rats. Acta Neuropathol..

[35] Alhilali LM, Delic JA, Gumus S, Fakhran S (2015). Evaluation of white matter injury patterns underlying neuropsychiatric symptoms after mild traumatic brain injury1. Radiology.

[36] Bramlett HM, Dietrich WD (2015). Long-term consequences of traumatic brain injury: Current status of potential mechanisms of injury and neurological outcomes. J. Neurotrauma.

[37] Kinnunen KM, Greenwood R, Powell JH, Leech R, Hawkins PC, Bonnelle V, Patel MC, Counsell SJ, Sharp DJ (2011). White matter damage and cognitive impairment after traumatic brain injury. Brain.

[38] Nennig SE, Schank JR (2017). The role of NFkB in drug addiction: Beyond inflammation. Alcohol Alcohol..

[39] Ahn HJ, Hernandez CM, Levenson JM, Lubin FD, Liou H-C, Sweatt JD (2008). c-Rel, an NF-κB family transcription factor, is required for hippocampal long-term synaptic plasticity and memory formation. Learn. Mem..

[40] Lu Y, Li T, Qureshi HY, Han D, Paudel HK (2011). Early growth response 1 (Egr-1) regulates phosphorylation of microtubule-associated protein tau in mammalian brain. J. Biol. Chem..

[41] Zalcman G, Federman N, de la Fuente V, Romano A (2015). Nuclear factor kappa B-dependent Zif268 expression in hippocampus is required for recognition memory in mice. Neurobiol. Learn. Mem..

[42] Gold EM, Vasilevko V, Hasselmann J, Tiefenthaler C, Hoa D, Ranawaka K, Cribbs DH, Cummings BJ (2018). Repeated mild closed head injuries induce long-term white matter pathology and neuronal loss that are correlated with behavioral deficits. ASN Neuro.

[43] Shen JX, Yakel JL (2009). Nicotinic acetylcholine receptor-mediated calcium signaling in the nervous system. Acta Pharmacol. Sin..

[44] Diaz-Arrastia R, Kochanek PM, Bergold P, Kenney K, Marx CE, Grimes Col JB, Loh LY, Adam LGE, Oskvig D, Curley KC, Salzer Col W (2013). Pharmacotherapy of traumatic brain injury: State of the science and the road forward: Report of the Department of Defense Neurotrauma Pharmacology Workgroup. J. Neurotrauma.

[45] Kochanek PM, Edward DC, Mondello S, Wang KKK, Lafrenaye A, Bramlett HM, Dietrich WD, Hayes RL, Shear DA, Gilsdorf JS, Catania M, Poloyac SM, Empey PE, Jackson TC, Povlishock JT (2018). Multi-center pre-clinical consortia to enhance translation of therapies and biomarkers for traumatic brain injury: Operation brain trauma therapy and beyond. Front. Neurol..

[46] Kochanek PM, Bramlett HM, Shear DA, Dixon CE, Mondello S, Dietrich WD, Hayes RL, Wang KKW, Poloyac SM, Empey PE, Povlishock JT, Mountney A, Browning M, Deng-Bryant Y, Yan HQ, Jackson TC, Catania M, Glushakova O, Richieri SP, Tortella FC (2016). Synthesis of findings, current investigations, and future directions: Operation brain trauma therapy. J. Neurotrauma.

[47] Morin A (2020). Therapeutic Target Identification, Validation and Drug Discovery for Traumatic Brain Injury.

