# Supplementary material for: A 3-month-delayed treatment with anatabine improves chronic outcomes in two different models of repetitive mild traumatic brain injury in hTau mice
Source: Sci Rep. 2021 Apr 12;11:7900. doi: 10.1038/s41598-021-87161-7 (PMC8041866; doi:10.1038/s41598-021-87161-7)
Supplement: Supplementary file 1 — Supplementary Figure S1. [file 41598_2021_87161_MOESM1_ESM.pdf]

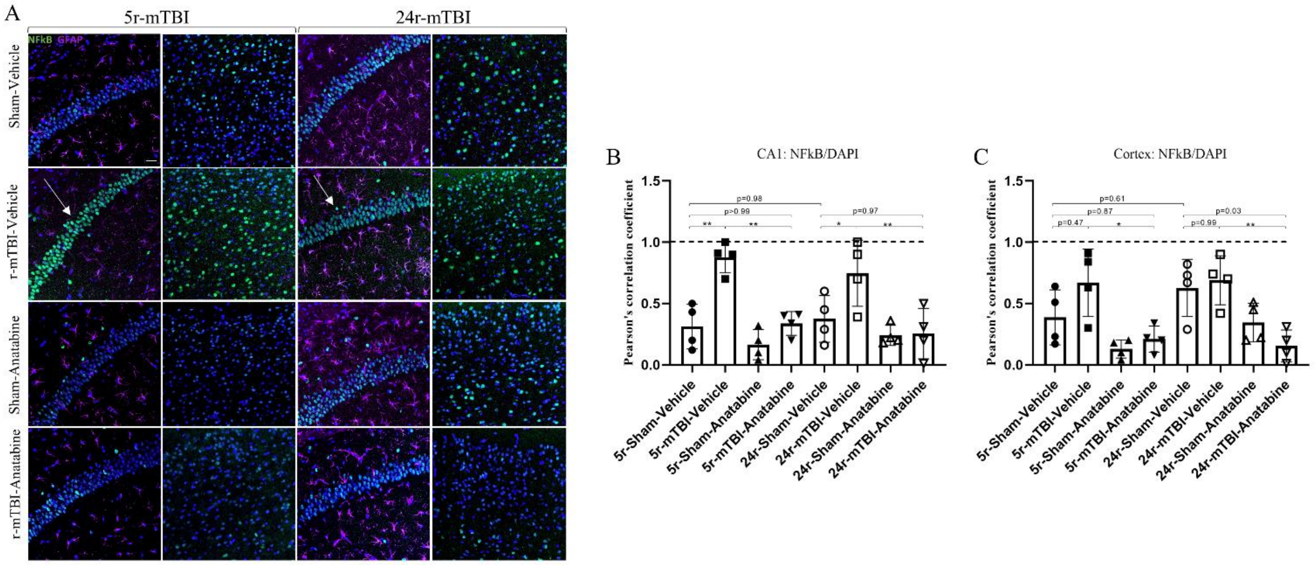

Supplementary Figure 1. Representative images of NFkB in cortex and CA1 area of hippocampus in 5r-mTBI and 24r-mTBI mice (A). Arrows indicate nuclear localization of NFkB. B-C – Quantitative analysis of NFkB/DAPI signal colocalization using Pearson's coefficient in indicate nuclear localization of NFkB. Scale bars equal 20µm.
